# Adverse drug reactions (ADRS) reporting: awareness and reasons of under-reporting among health care professionals, a challenge for pharmacists

**DOI:** 10.1186/s40064-016-3337-4

**Published:** 2016-10-12

**Authors:** Sumbul Shamim, Syed Muhammad Sharib, Saima Mahmood Malhi, Sidrat-ul Muntaha, Hassan Raza, Saniya Ata, Ali Salman Farooq, Mehwish Hussain

**Affiliations:** 1 Department of Pharmacology, Faculty of Pharmaceutical Sciences, Dow College of Pharmacy, Dow University of Health Sciences, Ojha Campus, Karachi, Pakistan; 2 Dow College of Pharmacy, Dow University of Health Sciences, Ojha Campus, Karachi, Pakistan; 3 Department of Research, Dow University of Health Sciences, Ojha Campus, Karachi, Pakistan

**Keywords:** Pharmacovigilance, ADR reporting, Awareness, Under reporting

## Abstract

**Objectives:**

To measure awareness about adverse drug reaction (ADRs) reporting among doctors, pharmacists and nurses and to determine reasons of ADRs under-reporting in Pakistan.

**Methods:**

In present study, a self-administered questionnaire was used to measure the awareness level about ADRs reporting among health care professionals (HCPs) of Pakistan. This was a cross sectional study.

**Results:**

Out of the respondents 51 % were physicians, 29.7 % pharmacists and 19.3 % were nurses. 65.5 % of HCP population observed ADRs, out of which only 57.4 % reported these in their respective hospitals. About 77.3 % of population understood the importance of reporting ADRs while 67.3 % of population agrees that pharmacists are chief personnel for the development of system. 71.8 % of HCPs agrees that ADRs are not reported because Community pharmacy lacks legally qualified pharmacists. Only 14.3 % of HCPs population knows that there is any ADR reporting organization in Pakistan.

**Conclusion:**

The study recommends the need of such reporting system and more than half of the studied population agreed that pharmacists are required in developing such system.

## Background

As per definition of Pharmacovigilance (PV) it is not only science but also actions which are for the detection, assessment, understanding and prevention of adverse effects or any other drug-related problem. The Thalidomide disaster in 1961 was the start of establishing the WHO Program for International Drug Monitoring, WHO promotes PV at the country level by working in collaboration with the Monitoring centre at Uppsala. More than 135 countries are the part of this program. This program not only enhances patient safety for use of medicines but also gives information about safe use and prevention and treatment of any Adverse Drug Reactions (ADRs) (http://www.who.int/medicines/areas/quality_safety/safety_efficacy/pharmvigi/en/).

WHO’s definition of ADRs, which has been in use for about 30 years, is “a response to a drug that is noxious and unintended and occurs at doses normally used in man for the prophylaxis, diagnosis or therapy of disease, or for modification of physiological function” (WHO 1972).

The birth defects were caused by thalidomide in 1961–1962 in about ten thousand children in different regions of the world, when the pregnant mothers used it for nausea and vomiting. As a result in 1968 the WHO started the Program for International Drug Monitoring (PIDM) for early detection of ADRs. This activity is now called as Pharmacovigilance. Uppsala Monitoring Centre (UMC) in Sweden is responsible to monitor and manage the WHO-PIDM activities (Collet 2000; Blenkinsopp et al. 2007; Gregg and Stuart 2010).

The inclination should be there to not only observe but also report unwanted and unexpected medical events in all areas where medicines are being used. At any dosage and by an overdose or by misuse or abuse of a medicine the adverse drug reactions or adverse events can occur (WHO 2002a). Pharmacovigilance is applied throughout the life cycle of a medicine that is from the pre-approval phase to the end use by the patients. These lead to burden on patients not only in disease form by prolonged stay in hospital but also the financial burden it creates immensely (Johnson and Bootman 1997). Pharmacovigilance focuses on not only effectiveness and benefits but also on safety and risk analysis with the aim to improve patient care (Cipolle et al. 2004; www.fda.com). The safety of patients is totally related with the safety of medicines. ADR monitoring is an integral part of quality assurance department in developed countries but unfortunately Pakistan has limited accountability system for medicines (Scurti et al. 2012; Rollins 2013).

Healthcare systems rely mainly on the detection and reporting of suspected ADRs to identify new reactions, record the frequency with which they are reported, evaluate factors that may increase risk and provide information to prescribers with a view to preventing future ADRs, shows that adverse drug reaction are by anyway causing deaths (Collet 2000). The actual statistics of ADR related death in Pakistan is not available because of underdevelopment of such system throughout Pakistan few of the hospitals like AGHA KHAN and DOW UNIVERSITY HOSPITAL practices reporting at their own level but system in just a few health care sectors in a large population is not sufficient obviously. Efforts are increasing to ensure that resource poor countries, which bear almost 90 % of the global disease burden, have access to effective medicines (Mahmood et al. 2011). Pakistan Pharmacist Federation has launched a campaign to implement the directions of Health Department, Government of Punjab, Pakistan to establish pharmacovigilance centre, adverse drug reporting, drug information and poison control centre at provincial and hospital level (Davies et al. 2009). Medication errors are usually not reported for the reason that the errors are considered as not very much significant by the prescriber that they should be reported (www.pharmacistfed.wordpress.com). Under reporting of ADRs influenced by prescriber’s and reporter’s medical knowledge and their approach to give significance to any types of ADR. This under reporting creates a negative impact on Public Health (Pirmohamed et al. 2007).

The purpose of the study is to evaluate the knowledge and concerns of the health care professionals about adverse drug reaction and its reporting which claims the development and incorporation of system including adverse drug reaction reporting and training to health care professionals about detection, assessment and control adverse drug reaction. Even though in countries like UK where pharmacovigilance activities are being practiced, the occurrence rate of ADR is 6.7 % with overall fatality rate is 0.32 % (Kazeem and Jacob 2009). Our study acknowledges the importance of ADR reporting and steps must be taken at national level to ensure the incorporation of pharmacovigilance centre in the health care sector.

## Method

### Design

A cross sectional study was conducted from June, 2013 to August, 2014 in Karachi, a metropolitan city of Pakistan. Target responders consisted of different health care professionals including nurses, pharmacist and physicians/doctors working in different health care services of the city.

The questionnaire being addressed was adapted from studies regarding concerns of health care professionals about adverse drug reaction reporting and reasons of underreporting these reactions, information and knowledge about reporting of ADR. Health care professionals mostly addressed were physicians, pharmacists and nurses working at public sector hospitals. (WHO Program for International Drug Monitoring 2010)

Institutional Research Review Committee has approved this study and has found it exempted from any IRB.

### Sample size

At 99 % confidence interval with 5 % bound of error of unawareness rate of 84 % of Irish physicians (Williams and Feely 1999). The calculated sample size was 357 HCPs which was calculated by the population size of HCPs in Karachi, Pakistan.

### Study population

According to sample size calculated 357 questionnaire forms were distributed to health care professionals of different Tertiary Health Care sectors of Karachi, including Dow University Hospital, Civil Hospital, Abbasi Shaheed Hospital, Liaquat National Hospital, Orthopedics and Medical Institute, Patel Hospital. Questionnaire forms were self addressed to HCPs.Of the respondents 51 % were physicians including house officers, RMO’s, surgeons, consultant doctors, and 29.7 % were pharmacist working at inpatient pharmacy services and 19.3 % nurses.

### Statistical analysis

Data were analyzed using Statistical Package for Social Sciences (SPSS) version 21. Descriptive statistics were employed to report the response of respondents in terms of frequency and percentage. Since, responses were ordinal scaled, therefore, gradient effect Chi-square test was executed to measure association of knowledge, attitude, perception, practices and reasons of non-compliance with different HCPs. P value less than 0.05 was considered to show significant association.

## Results

A total of 357 responses were compiled in the data file. Out of the respondents 51 % (n = 182) were physicians, 29.7 % (n = 106) were pharmacists and 19.3 % (n = 69) were nurses. The findings depicted only 43.4 % (n = 155) HCPs knew the term Pharmacovigilance and ADR reporting. The frequency of knowledge of term Pharmacovigilance was significantly more among pharmacists followed by physicians (P < 0.0001). Only 31.7 % of respondents know that there is any ADR reporting form at the website of Drug Regulatory Authority of Pakistan (DRAP) while DRAP is established since 2012. Pharmacist followed by nurses confirmed the knowledge of DRAP webpage significantly more than physicians (P = 0.017). Furthermore only 14.3 % of HCPs respondents knows that there is any ADR reporting organization in Pakistan. Though, the linear association of the knowledge of ADR reporting organization in Pakistan was not significant showing similar knowledge among three types of HCPs (Table 1) indicating that there is a problem about national reporting in Pakistan across all the professionals.

**Table 1:** Knowledge about ADR reporting from three different HCPs

	Designation						P value
	Physician		Pharmacist		Nurse		
	N	%	N	%	N	%	
I know the term pharmacovigilence and ADR reporting							
Strongly disagree	7	3.8	0	0.0	0	0.0	<0.0001
Disagree	10	5.5	0	0.0	3	4.3	
Neutral	116	63.7	29	27.4	37	53.6	
Agree	33	18.1	31	29.2	26	37.7	
Strongly agree	16	8.8	46	43.4	3	4.3	
I know that there is a form for ADR reporting available at ministry of health (DRAP) website							
Strongly disagree	25	13.7	2	1.9	10	14.5	0.017
Disagree	83	45.6	23	21.7	26	37.7	
Neutral	37	20.3	22	20.8	16	23.2	
Agree	25	13.7	38	35.8	13	18.8	
Strongly agree	12	6.6	21	19.8	4	5.8	
I know the ADR reporting organization where I can report, in pakistan							
Strongly disagree	12	6.6	1	0.9	5	7.2	0.937
Disagree	32	17.6	26	24.5	21	30.4	
Neutral	122	67.0	51	48.1	36	52.2	
Agree	9	4.9	11	10.4	5	7.2	
Strongly agree	7	3.8	17	16.0	2	2.9	

About 67.3 % of respondents agreed that pharmacists are chief personnel for the development of system (Table 2). The agreement of relating pharmacists as chief personnel for reporting ADR was proportionally least among physicians followed by nurses (P = 0.0002). The proportion of agreement to collaborate pharmacist with other HCPs was high among pharmacist. The requirement of drug utilization review and ADR reporting system is highly acknowledged by all three health care professionals (P = 0.308).

**Table 2:** Attitude for ADR reporting from three different HCPs

	Designation						P value
	Physician		Pharmacist		Nurse		
	N	%	N	%	N	%	
Pharmacists are the cheif personel required for ADR reporting system development							
Strongly disagree	14	7.7	2	1.9	0	0.0	0.002
Disagree	22	12.1	8	7.5	8	11.6	
Neutral	37	20.3	11	10.4	15	21.7	
Agree	75	41.2	43	40.6	25	36.2	
Strongly agree	34	18.7	42	39.6	21	30.4	
Other health care professionals must be in collaboration of pharmacists, physicians and nurses							
Strongly disagree	4	2.2	0	0.0	1	1.4	0.068
Disagree	13	7.1	2	1.9	4	5.8	
Neutral	24	13.2	7	6.6	7	10.1	
Agree	90	49.5	43	40.6	37	53.6	
Strongly agree	51	28.0	54	50.9	20	29.0	
Drug utilization review and ADR reporting system is required in our hospitals to reduce adverse drug events							
Strongly disagree	6	3.3	1	0.9	0	0.0	0.308
Disagree	9	4.9	1	0.9	4	5.8	
Neutral	39	21.4	9	8.5	19	27.5	
Agree	84	46.2	47	44.3	33	47.8	
Strongly agree	44	24.2	48	45.3	13	18.8	

About 77.3 % of respondents understood the importance of reporting ADRs. Though, only 38.9 % confessed presence of ADR reporting system in their respective health care system (Table 3). There was no significant association of owning the responsibility of ADR reporting among these three professionals. While asking about presence of ADR reporting system in respective health care setup, the proportion of denying the same was significantly higher from physicians, followed by pharmacists (P < 0.0001).

**Table 3:** Perception about ADR reporting from three different HCPs

	Designation						P value
	Physician		Pharmacist		Nurse		
	N	%	N	%	N	%	
ADR reporting is my responsiblity							
Strongly disagree	6	3.3	2	1.9	1	1.4	0.986
Disagree	18	9.9	3	2.8	9	13.0	
Neutral	24	13.2	6	5.7	12	17.4	
Agree	78	42.9	38	35.8	35	50.7	
Strongly agree	56	30.8	57	53.8	12	17.4	
ADR reporting system is present in my health care sector setup							
Strongly disagree	39	21.4	3	2.8	5	7.2	<0.0001
Disagree	25	13.7	16	15.1	7	10.1	
Neutral	73	40.1	32	30.2	18	26.1	
Agree	15	8.2	28	26.4	22	31.9	
Strongly agree	30	16.5	27	25.5	17	24.6	

Nearly half percent (n = 186) HCPs were found to be trained for detecting, reporting and controlling ADR. The training of detection and reporting of ADR was found significantly more among nurses (P = 0.006). Around 65.5 % of HCP respondents observed ADR whereas only 57.4 % report these in their respective hospitals. Observation of ADR was significantly not different among these three HCPs. Though, reporting the same was found significantly least among physicians followed by nurses (P = 0.001). Reporting of ADR to any pharmaceutical industry was not in higher proportion (Table 4).

**Table 4:** Practices for ADR reporting from three different HCPs

Practices	Designation						P value
	Physician		Pharmacist		Nurse		
	N	%	N	%	N	%	
I am trained to detect, report and control ADR							
Strongly disagree	14	7.7	2	1.9	2	2.9	0.006
Disagree	57	31.3	12	11.3	11	15.9	
Neutral	35	19.2	20	18.9	18	26.1	
Agree	45	24.7	46	43.4	34	49.3	
Strongly agree	31	17.0	26	24.5	4	5.8	
I have observed ADR							
Strongly disagree	11	6.0	1	0.9	1	1.4	0.29
Disagree	32	17.6	7	6.6	12	17.4	
Neutral	27	14.8	19	17.9	13	18.8	
Agree	71	39.0	39	36.8	34	49.3	
Strongly agree	41	22.5	40	37.7	9	13.0	
I report ADR in my health care sector							
Strongly disagree	6	3.3	2	1.9	2	2.9	0.001
Disagree	52	28.6	9	8.5	4	5.8	
Neutral	35	19.2	25	23.6	17	24.6	
Agree	57	31.3	44	41.5	34	49.3	
Strongly agree	32	17.6	26	24.5	12	17.4	
I report ADR in any pharmaceutical industry							
Strongly disagree	18	9.9	9	8.5	8	11.6	0.17
Disagree	85	46.7	44	41.5	23	33.3	
Neutral	46	25.3	24	22.6	22	31.9	
Agree	30	16.5	26	24.5	15	21.7	
Strongly agree	3	1.6	3	2.8	1	1.4	

Furthermore, our study also highlighted various reasons of underreporting. HCPs (84.6 %) have uncertainty whether the ADRs occurred due to drugs, unavailability of reporting forms (e.g. yellow cards), 71.8 % of HCPs agrees that ADRs are not reported because Community pharmacy lacks legally qualified pharmacists (Table 5). Physicians were significantly least agreed of ADR underreporting due to shortage of time (P = 0.013), complacency (P = 0.011) and belief of safe marketed drugs (P = 0.006).

**Table 5:** Reasons for ADR misreporting from three different HCPs

Reasons	Designation						P value
	Physician		Pharmacist		Nurse		
	N	%	N	%	N	%	
ADR reporting system not incorporated because of limited awareness of the health care professionals							
Strongly disagree	7	3.8	0	0.0	1	1.4	0.515
Disagree	16	8.8	8	7.5	12	17.4	
Neutral	42	23.1	11	10.4	22	31.9	
Agree	76	41.8	49	46.2	23	33.3	
Strongly agree	41	22.5	38	35.8	11	15.9	
ADR reporting system not incorporating in Karachi Hospitals because of financial issues							
Strongly disagree	10	5.5	2	1.9	3	4.3	0.982
Disagree	37	20.3	18	17.0	14	20.3	
Neutral	46	25.3	26	24.5	17	24.6	
Agree	58	31.9	44	41.5	30	43.5	
Strongly agree	31	17.0	16	15.1	5	7.2	
ADR’s not reported because of shortage of time							
Strongly disagree	10	5.5	2	1.9	1	1.4	0.013
Disagree	49	26.9	21	19.8	13	18.8	
Neutral	40	22.0	14	13.2	17	24.6	
Agree	56	30.8	42	39.6	25	36.2	
Strongly agree	27	14.8	27	25.5	13	18.8	
ADR’s not reported because of unavailiblity of reporting forms							
Strongly disagree	8	4.4	1	0.9	0	0.0	0.517
Disagree	26	14.3	22	20.8	11	15.9	
Neutral	40	22.0	19	17.9	17	24.6	
Agree	84	46.2	50	47.2	30	43.5	
Strongly agree	24	13.2	14	13.2	11	15.9	
ADR’s not reported because complacency (uncertinity that ADR occurred due to drug administered)							
Strongly disagree	4	2.2	3	2.8	0	0.0	0.011
Disagree	22	12.1	14	13.2	7	10.1	
Neutral	127	69.8	44	41.5	44	63.8	
Agree	22	12.1	34	32.1	12	17.4	
Strongly agree	7	3.8	11	10.4	6	8.7	
ADR’s not reported because of the belief that all marketted drugs are safe							
Strongly disagree	13	7.1	15	14.2	1	1.4	0.006
Disagree	35	19.2	27	25.5	14	20.3	
Neutral	122	67.0	48	45.3	35	50.7	
Agree	12	6.6	10	9.4	13	18.8	
Strongly agree	0	0.0	6	5.7	6	8.7	
Community pharmacy (medical stores) lacking legally qualified pharmacist causing non reporting							
Strongly disagree	7	3.8	3	2.8	0	0.0	0.243
Disagree	26	14.3	1	0.9	7	10.1	
Neutral	27	14.8	13	12.3	17	24.6	
Agree	60	33.0	30	28.3	28	40.6	
Strongly agree	62	34.1	59	55.7	17	24.6	
Fatal ADR’s are mostly not reported because usually general public donot allow conducting postmartem studies							
Strongly disagree	5	2.7	0	0.0	0	0.0	0.721
Disagree	20	11.0	4	3.8	3	4.3	
Neutral	24	13.2	18	17.0	18	26.1	
Agree	75	41.2	46	43.4	34	49.3	
Strongly agree	58	31.9	38	35.8	14	20.3	

## Discussion

Because of a counterfeit antihypertensive medicine at the Punjab Institute of Cardiology (PIC) hospital at Lahore, Pakistan, the lives of over 100 heart patients were taken away in January 2012 (Kazeem and Jacob 2009). The counterfeit medicine(s) deposited in the bone marrow of patients and lost the patient’s resistances. The drug caused the suppression of generation of white blood cells after being deposited in bone marrow. Which lead to change in skin pigmentation, severe chest infection, decreased platelet count and blood vomiting (Thenews.com.pk 2012).

As it has been reported that the infer drugs include isotab (isosorbide nitrate), lipitor (atorvastatin calcium), soloprin (aspirin), cardiovestin (simvastatin), concort (amlodipine), and alfagril (clopidogrel) (Desk 2012).

Import of medicines from Pakistan was banned by Sri Lanka as a precautionary measure (Usman 2012). As a consequence, Efroze Pharma was called by the WHO for increased vigilance on the use of isotab, for which it released a global drug safety alert (no. 125) (Wasif 2012).

Considering the importance of ADRs reporting, our study showed inadequate knowledge of physician and nurses about an adverse drug reaction and reporting. Study also reveals the point of enhancing awareness among the HCPs also increase in DRAP’s role for this purpose. (WHO 2002b)

Doctors, pharmacists and nurses as well as patients can report ADRs. This may become more rapid and advanced by the use of new softwares and internet. The way in which companies and governments handle patients data may become safe by law now is passed by the member states will enable the security and privacy of patient data (The News Tribe 2012; MedWatch 2015; Chanda 2007; Strengthening Pharmaceutical Systems (SPS) 2009; Cobert and Silvey 1999).

Because of absence of appropriate clinical trials in the paediatric population, drugs prescription in children has a high risk of developing unknown or rare adverse drug reactions (ADRs). The spontaneous reporting of suspected ADRs is an important way to promote reasonable warning signals. The family paediatricians (FPs) play a crucial role in this reporting (http://www.isoponline.org).

Pharmacists can be the chief personnel for the development of Pharmacovigilance system as 59.89 % (n = 109) Physicians, 66.66 % (n = 46) nurses and 80.1 % (n = 85) Pharmacists agrees upon the point. In 60.1 % of HCPs opinion there is no any Pharmacovigilance system exists in their respective health care sectors.

As per study results, HCPs observe ADRs during their clinical practice but the reporting of those ADRs are very much limited to the concerning authorities whether Pharmaceutical Industry or Government concerned department (Khurshid et al. 2008).

Furthermore, study reveals the reasons that could be looked into for the betterment of reporting of ADRs. According to the study, reasons mainly causing underreporting of ADR’s are, Uncertainty of whether ADRs occur due to drug or not, community pharmacy lacking legally qualified pharmacist, Unavailability of ADR reporting form (e.g. Yellow cards), and awareness regarding system (Pellegrino et al. 2013). Financial issues can be resolved if the steps are taken at governmental level. Physicians generally are not very much responsive to ADR reporting program mainly because of the time that they prefer to reserve for patients rather to spare time for reporting (Table 5). Other reasons also contributes but to a lesser extent in underreporting of ADRs (WHO 2002b).

Study also force on the point of having Drug Utilization Review or ADR reporting system within the Health Care Sectors to reduce the rate of adverse drug events (http://www.who-umc.org).

Incorporation of pharmacists in health care sectors for making policies regarding the system and acceptability among other health care professionals for the pharmacists to detect report and control ADRs is necessary. As has been mentioned that physicians are not very cooperative to report ADRs so, to increase reporting many countries allowed pharmacists working in hospital and community, nurses and even patients to report ADR (Olsson 1998).

There is also presence of nurses throughout in all hospitals especially in public sectors hospitals who even do not know about the presence of such reactions even most of the At least 60 % of ADRs are preventable (http://www.isoponline.org).

Medical practitioners are the primary component of ADR reporting system but every healthcare professional who is having knowledge, attitudes and perceptions about ADR can play its part in reporting ADRs (Khurshid et al. 2008). As 77.47 % (n = 141) Physicians, 82.01 % (n = 57) nurses and 91.51 % (n = 97) pharmacists agrees upon the point (Table 2).

## Conclusion

Adverse drug events are preventable most of the time. Even then it is reported to be the 8^th^-leading cause of death which exceeds the deaths attributable to motor vehicle accidents, breast cancer or AIDS (Shin et al. 2011). These results shows that even in developed country where expertise are practicing such system, the ADR related deaths are a matter of serious concerns.

Pharmacovigilance system implementation is the need which is possible by collaboration between academia, health care providers including pharmacist, patient, manufacturer, government, media, and civil society, Uppsala Monitoring Center (UMC), Sweden operating under (WHO), FDA, Isop and other international organization working on drug safety. To enhance the patients trust it is great opportunity for Pharmacists to built Pharmacovigilance system in Pakistan (http://www.ppma.org.pk/PPMAIndustry.aspx). Reporting of any doubtful ADR event by health care professional is of concern because seemingly the relationship between the medicine and its reaction is not very much clear but analysis after reporting can evaluate its importance. Further, such PVsystem on national level can work with help of coordinator and a core committee to make plans and to take decision for the national centre to maintain the quality (National Health Policy 2001). Focus group interviews can be beneficial retrieving the reasons of underreporting than deliberately action upon them can lead to appropriate reporting leading to better patient health care (http://www.dcomoh.gov.pk/). Fear of reaction from the nurse managers and coworkers, fear of termination from job are found to be the reasons of underreporting of medication errors by nurses. With the help of continuous medical education programs the nurses knowledge regarding medications can be enhanced (http://www.pharma-iq.com/regulatorylegal/articles/building-a-harmonisedpharmacovigilanceframework/).
